# Ethanol extract of *Portulaca oleracea* L. mitigates atherosclerosis through modulation of cholesterol efflux and uptake pathways

**DOI:** 10.3389/fphar.2025.1550812

**Published:** 2025-03-19

**Authors:** Bin Chen, Shanshan Sun, Jialei Fu, Ling Ge, Wei Nie, Peina Zhou, Peng Cao, Qian Zhou

**Affiliations:** ^1^ Affiliated Hospital of Integrated Traditional Chinese and Western Medicine, Nanjing University of Chinese Medicine, Nanjing, China; ^2^ Institute of Plant Resources and Chemistry, Nanjing Research Institute for Comprehensive Utilization of Wild Plants, Nanjing, China; ^3^ State Key Laboratory of Materials-Oriented Chemical Engineering, College of Food Science and Light Industry, Nanjing Tech University, Nanjing, China; ^4^ State Key Laboratory on Technologies for Chinese Medicine Pharmaceutical Process Control and Intelligent Manufacture, Nanjing University of Chinese Medicine, Nanjing, China

**Keywords:** *Portulaca oleracea*, bioactive ingredients, atherosclerosis, cholesterol metabolism, reverse cholesterol transport

## Abstract

**Background:**

Purslane (*Portulaca oleracea*) is a medicinal and edible plant. Purslane extract (POEE) exhibits lipid-lowering, anti-inflammatory, and antioxidant properties. Traditionally, this extract has been used to treat various inflammatory conditions, including skin inflammation, enteritis, and dysentery. However, its therapeutic potential and molecular mechanisms in atherosclerosis (AS) remain unclear.

**Methods:**

Ultra-performance liquid chromatography-quadrupole/time-of-flight mass spectrometry (UPLC-Q/TOF-MS) and the Traditional Chinese Medicine Systems Pharmacology Database were employed to identify the active components of POEE. Network pharmacology was used to predict POEE’s mechanisms for alleviating AS. An *in vitro* foam cell model was established by treating RAW264.7 macrophages with oxidized low-density lipoprotein (ox-LDL), and the protective effects of POEE were assessed via the 3-[4,5-dimethylthiazol-2-yl]-2,5 diphenyl tetrazolium bromide (MTT) assay, while intracellular lipid accumulation was identified using Oil Red O staining. Protein expression related to cholesterol metabolism was analyzed by Western blot (WB). For *in vivo* validation, AS was induced in rats through a high-fat diet and carotid artery injury. After 4 weeks of daily POEE administration, the therapeutic efficacy was tested by measuring serum lipid levels, cardiac function, histopathological changes, and the cholesterol transport-related protein expression.

**Results:**

The bioactive compounds identified in POEE were categorized into 10 groups, including flavonoids (24), terpenoids (16), phenols (6), and alkaloids (4), and others. Network pharmacology predictions implicated POEE in modulating the “Lipid and Atherosclerosis” pathway. POEE significantly reduced total cholesterol (TC) and free cholesterol (FC) levels in ox-LDL-stimulated macrophages, attenuating foam cell formation. Furthermore, POEE enhanced reverse cholesterol transport (RCT) by upregulating the expressions of ATP-binding cassette transporters ABCA1 and ABCG1 to promote cholesterol efflux, while suppressing CD36 and MSR1 expressions to inhibit cholesterol uptake. *In vivo*, POEE administration lowered serum triglycerides (TG), TC, FC, and LDL-C levels; elevated HDL-C; and ameliorated carotid artery lesions in AS rats. Concordantly, ABCA1 expression was upregulated and that of MSR1 was downregulated in POEE-treated carotid tissues.

**Conclusion:**

POEE alleviates atherosclerosis by enhancing RCT through regulation of cholesterol efflux and uptake pathways. POEE may be a promising therapeutic candidate for AS.

## 1 Introduction

Atherosclerosis (AS) is a chronic inflammatory disorder involving lipid accumulation, inflammation, cellular death, and fibrosis in the arterial tunica intima (Williams et al., 2020). Currently, the primary mechanisms of prevention and management of AS focus on lipid regulation, anti-inflammation, and antihypertensive control (Wang et al., 2019; Devesa et al., 2023). Central to AS pathogenesis is foam cell formation—a consequence of dysregulated cholesterol homeostasis involving influx, esterification, and efflux imbalances (Ren et al., 2021). The hallmark pathological feature of AS is lipid-rich plaques, characterized by abundant macrophage-derived foam cells within the arterial intima (Zhao et al., 2019). Conversely, reverse cholesterol transport (RCT) exported excess cholesterol from foam cells to the liver for conversion into bile acids and subsequent fecal excretion (Chen et al., 2023). Thus, RCT represents a critical and potentially pivotal defense mechanism in mitigating the progression of AS.

RCT is a cholesterol metabolism pathway, and cholesterol efflux can remove free cholesterol from macrophages through active transfer or passive transmembrane diffusion mediated by cholesterol transporters (Chen et al., 2023). Subsequently, HDL or apolipoprotein (Apo) A1 captures and releases cholesterol. The cholesterol transporters, ATP-binding cassette (ABC) transporters (ABCA1 and ABCG1), play major roles in active free cholesterol efflux (Bi et al., 2017). Macrophage scavenger receptor 1 (MSR1) and cluster of differentiation 36 (CD36) are scavenger family A and B receptor proteins, respectively (Bieghs et al., 2010). They are the primary receptors for phagocytosis and uptake of oxidized low-density lipoprotein (ox-LDL) by macrophages cells, accounting for 90% of the ox-LDL load of macrophages. This dynamic imbalance between ABCA1-/ABCG1-facilitated cholesterol export and CD36-/MSR1-dependent ox-LDL uptake constitutes a pivotal pathological mechanism driving foam cell transformation and atherosclerotic plaque progression (Duan et al., 2017).

**FIGURE 1:**
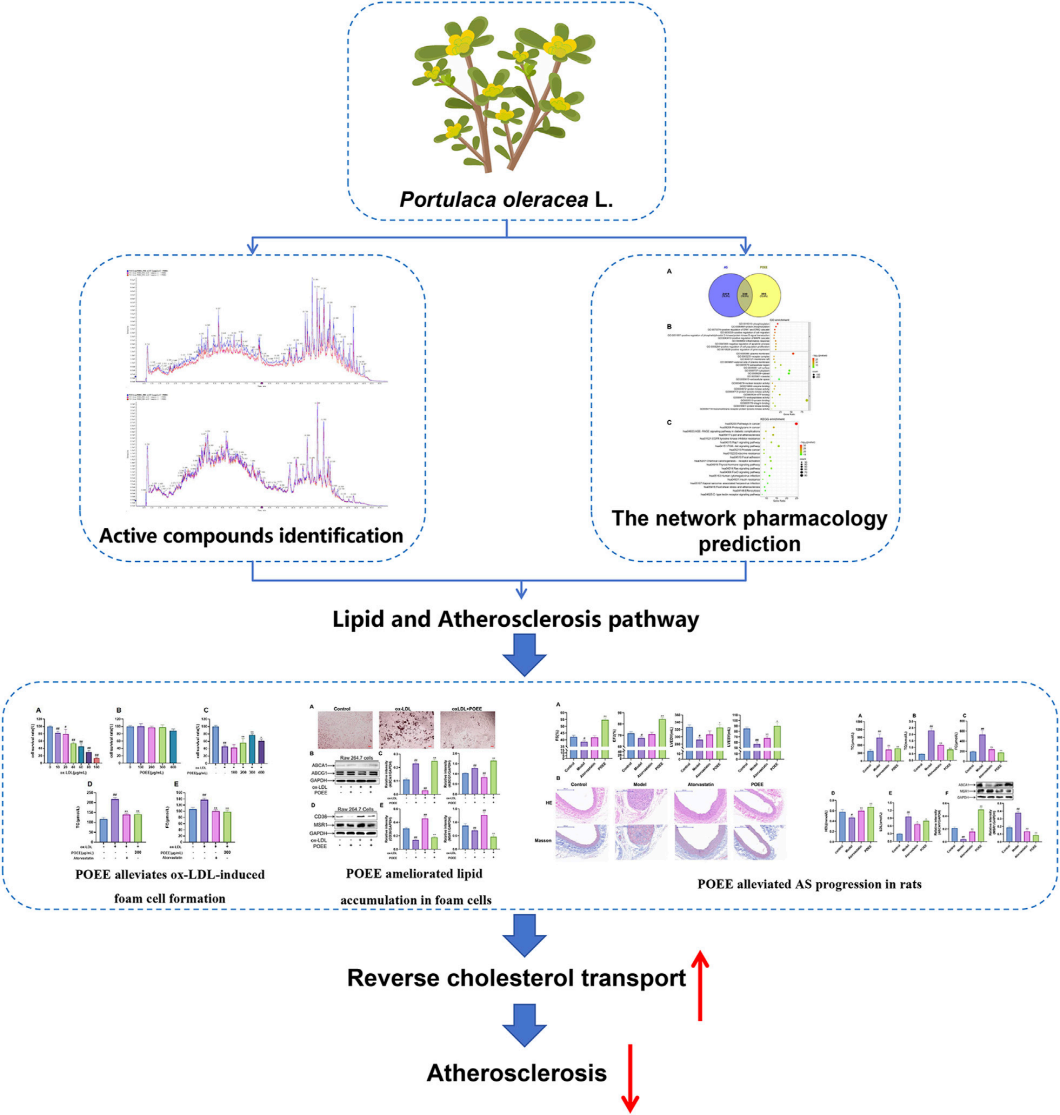
Flowchart of this study.

*Portulaca oleracea,* widely distributed in temperate and tropical regions, is recognized by the World Health Organization as one of the most extensively used medicinal plants and a “global panacea*”* (Zhang et al., 2020). Its phytochemical components include flavonoids, terpenoids, alkaloids, coumarin, organic acids, and polysaccharides (Li et al., 2024). These bioactive constituents exhibited multi-target effects, including anti-inflammatory, anti-tumor, and antimicrobial activities and metabolic management (Gao et al., 2020; Park et al., 2019; Noorbakhshnia and Karimi-Zandi, 2017). The traditional use of *P. oleracea* extract (POEE) was in treating inflammatory disorders including skin inflammation, enteritis, and dysentery (Li et al., 2024). Emerging evidence supports that the *P*. *oleracea* plant has anti-atherosclerotic potential (Wang et al., 2024; Hao et al., 2024). Experimental studies demonstrated that the stem extract of *P. oleracea* has protective effects on hyperlipidemia (El-Newary, 2016). In addition, *Portulaca grandiflora,* the plant belonging to the same family as *P. oleracea*, played an important role in attenuating atherosclerotic lesion progression in experimental models. However, the precise mechanisms underlying POEE action have not yet been fully elucidated. Therefore, the objective of the current study was to explore the potential role of POEE against AS, with particular emphasis on its modulation of RCT pathways. The flow chart of this study is shown in Figure 1.

## 2 Materials and methods

### 2.1 Chemicals and reagents

DMEM-high glucose culture medium was purchased from American Hyclone Inc., and fetal bovine serum was purchased from Gibco Co. (United States). Ox-LDL was obtained from Guangdong YiYuan Biotech Co. Ltd. (China) (Liu et al., 2014). Shanghai Mclean Biochemical Technology Co. Ltd. (China) supplied the cell lysis buffer used for Western blot and immunoprecipitation (IP). Oil Red O dye was purchased from Sigma (United States). The TC and free cholesterol (FC) detection kits were purchased from Shanghai Yuan Ye Biotechnology Co. Ltd. The antibodies against ABCA1, ABCG1, CD36, and MSR1 were bought from Abcam Company (United States). Rutin (purity >98.0%), matrine (purity >98.0%), and glutamic acid (analytically pure) standards were purchased from Beijing Solarbio Technology Co. Ltd., Dalian Meilun Biotechnology Co. Ltd., and Sinopharm Chemical Reagent Co. Ltd., respectively.

### 2.2 Preparation of POEE

Manual crushing and high-speed grinding created a fine powder. The powder was sifted with a 40-mesh sieve. Based on the extraction optimization process of POEE (Kong et al., 2018), extraction was performed at the temperature of 50°C; 70% ethanol was used, with an extraction interval of 53 min and a solid-to-liquid ratio of 1:15 (g/mL). POEE was obtained after filtrate drying and volume confirmation. The concentration of the purslane extract was prepared according to the mass concentration required for the test.

### 2.3 Content determination of active fractions in POEE

#### 2.3.1 Determination of the total flavonoid content in POEE

Two milliliters of POEE followed by 0.5 mL of 5% sodium nitrite was pipetted into a volumetric flask, shaken, and left to stand. After that, 0.5 mL of 10% aluminum nitrate was added and mixed. At the end of the reaction, 5 mL of 4% sodium hydroxide was added to the flask and was filled to volume. After mixing, the solution was left undisturbed for 20 min, which led to the formation of the POEE reaction mixture (Yu et al., 2007). Using rutin as the standard, the total flavonoid content was assessed by measuring the mixture’s absorbance at 510 nm (Wang et al., 2012).

#### 2.3.2 Determination of the total alkaloid content in POEE

The content of total alkaloid in POEE was determined according to Gan et al. (2010). Matrine was conformed as the standard to measure the total alkaloid content.

#### 2.3.3 Determination of the total amino acid content in POEE

The content of total amino acid in POEE was determined according to Wang (2006). In addition, the total amino acid content was measured using glutamic acid as the standard.

#### 2.3.4 Chemical profiling of POEE based on UPLC-Q-TOF-MS/MS

The parameter setting of UPLC-Q-TOF-MS/MS was referred to in Zhang C. et al. (2023). The sample injection volume was 2 μL.

A comprehensive score of 0.7 or more was used to identify the compounds in POEE by comparing the retention time, molecular weight (with an error of less than 10 ppm), secondary fragmentation spectra, collision energy, and other details. The methods employed for identification and statistical analysis of the compounds were comparable to those used in Fei et al. (2022).

### 2.4 Network pharmacology analysis

#### 2.4.1 Screening of active components

All identified POEE components were input into the TCMSP as references (Ru et al., 2014; Zhang J. et al., 2023). The screening criteria were as follows: oral bioavailability (OB) ≥ 20%, human Caucasian colon adenocarcinoma (Caco-2) ≥ – 0.4, and drug-likeness (DL) ≥ 0.18. Compounds that meet these requirements are considered to be active components. In addition, as reported in literature, some compounds with pharmacological activities do not meet the above criteria were included in the scope.

#### 2.4.2 Mining of targets for AS treatment with POEE

To discover the therapeutic targets for AS treatment with POEE, the keyword “atherosclerosis” was used to mine relevant target genes from databases (GeneCards, OMIM, DrugBank, and DisGeNET). In addition, gene expression data for AS (GSE100927) were downloaded from the Gene Expression Omnibus (GEO) database (https://www.ncbi.nlm.nih.gov/geo/) for statistical analysis. The selection criteria included the following: GeneCards score ≥1 and DisGeNET score ≥0.02, and for GEO analysis, *p* < 0.05 and |logFC| > 1. After combining all the target genes from the sources mentioned above, duplicate targets were eliminated. Furthermore, the active components of POEE were imported into SwissTargetPrediction (http://www.swisstargetprediction.ch/).

The target gene identification can be referred to in Bardou et al. (2014); Daina and Zoete (2024). Gene symbols were used to standardize the target genes using the UniProt database (https://www.uniprot.org/). We used Venny 2.1 (https://bioinfogp.cnb.csic.es/tools/venny/) to find these genes’ intersections, which were considered targets for POEE in treating AS (Bardou et al., 2014).

#### 2.4.3 GO and KEGG enrichment analysis

The identified target genes of GO and KEGG enrichment were referred to in Fei et al. (2022). A *p*-value of less than 0.01 was used to screen and present the top 10 GO terms and the top 20 KEGG pathways.

### 2.5 Cell viability and cell proliferation assays

The methods for cell culture of RAW264.7 cells were referred to in Picard et al. (2011). Cells at different densities were cultured using the 3-[4,5-dimethylthiazol-2-yl]-2,5 diphenyl tetrazolium bromide (MTT) assay (6 × 10^3^ cells/well), Oil Red O staining (4 × 10^4^ cells/well), lipid metabolism index detection (1 × 10^5^ cells/well), and Western blot.

RAW264.7 cells were prepared in high-glucose Dulbecco's Modified Eagle's Medium (DMEM) supplemented with 10% fetal bovine serum (FBS), partial shipments in 96-well plates, each group of five holes, 100 μL per hole, at 37°C, and 5% CO_2_ incubator adaptability training in 24 h. We added 20, 40, 60, 80, and 100 μg/mL of ox-LDL to the culture medium for 24-h incubation to induce the foam cells. Subsequently, POEE (final concentrations of 100, 200, 300, and 400 μg/mL) was added to foam cells, followed by the replacement of serum-free culture, with each hole containing 100 μL, as determined by MTT. After 2 h, the medium was drained, and 100 μL of dimethyl sulfoxide (DMSO) was added. The plates were shaken and incubated at 37°C for 10 min, and the absorbance was measured at 450 nm wavelength. The cell viability was calculated based on the absorbance values of each group.

According to the above results, 60 mg/L ox-LDL was added with POEE (final concentration of 300 μg/mL) to the cells for protection and intervention for 4 h. The serum-free medium was replaced, and 100 μL of MTT was added to each well. The liquid was dried up after 2 h, and 100 μL of DMSO was added. Furthermore, the plates were shaken and incubated at 37°C for 10 min, and the absorbance was measured at 450 nm wavelength. The cell viability was calculated based on the absorbance observed in each group.

### 2.6 Oil Red O staining

The RAW264.7 cells in the DMEM containing 10% fetal bovine serum were used for cell adhesion and plated in a 24-well culture plate. They were divided into the blank control group, model group, and POEE dosage groups, at a density of 3 × 10^5^ cells/mL, adaptability training after 24 h, suck out the culture, and PBS washing twice each every hole to join 500 μL DMEM medium sugar, to medicine group to join the final concentration of POEE (300 μg/mL). Following a 4-h incubation, 60 μg/mL of ox-LDL was successfully added to the model group to commence modeling. After 24 h of reincubation and washing with PBS twice, “oil red O” staining was performed, and the staining effect between each group was observed under a microscope.

### 2.7 Animals

This study was approved by the Animal Ethics Committee of Nanjing University of Chinese Medicine. We used 40 healthy Sprague–Dawley (S-D) male rats aged 7–8 weeks and weighing 180∼220 g (License No. SCXK (Jing) 2019–0016). All animal experiments in this study were approved by the Ethics Committee of Jiangsu Province Hospital on Integration of Chinese and Western Medicine (AEWC-20200518-106). The rats were maintained as mentioned in Duan et al. (2024). Following 1 week of adaptation, the rats were randomly divided into four groups: control, model, positive control group (5 mg/kg/day), and the POEE group (1.0 g/kg/day), and they were fed adaptively for 1 week. The control group was fed a normal diet, whereas the other groups were fed a high-fat diet (HFD). An intraperitoneal dose of 400,000 IU/kg of vitamin D3 injection was administered. Carotid artery injury was performed 3 weeks later. In 4 weeks, the drug was administered by oral gavage at the corresponding dose. The blank control group was given a constant volume of normal saline once daily for weeks. The mental state and diet were monitored daily, whereas the weight was recorded weekly.

All operations were performed under 1% isoflurane anesthesia. The rats were placed in the anesthetic box, after which they were placed on the test table (temperature was set at 37°C) after anesthesia. Breathing masks were put on them to maintain the anesthetic state. The skin of the rat’s chest was depilated, a coupling agent was applied, and the VEVO 3100 ultrasound for small animals was used for transthoracic echocardiography. Indicators related to cardiac function in the left ventricle of the rats were detected using a 400× probe. The long and short axes of the left ventricle of the rats were detected by adjusting the probe’s position, the B- and M-mode images were collected, and the original data were recorded.

### 2.8 Biochemical analysis

The levels of TG, TC, FC, HDL, and LDL in rat serum and TC and FC in the foam cells were determined using the biochemical kit, according to the manufacturer’s instructions.

### 2.9 HE staining and Masson staining

The carotid artery of each group was fixed with 10% formalin, embedded in paraffin, sliced to 5 μm thickness, stained with HE and Masson stain, and observed under a light microscope (Leica DM3000, Germany).

### 2.10 Western blot analysis

The cells and tissues for WB can be referred to in Yu et al. (2020). The primary antibodies included glyceraldehyde 3-phosphate dehydrogenase (GAPDH), ABCA1, ABCG1, CD36, and MSR1. Protein bands were visualized using the ChemiDoc imaging system (Bio-Rad Laboratories, Berkeley, CA, United States).

### 2.11 Statistical analysis

All data were presented as mean ± standard deviation (SD). GraphPad Prism 9.0 (GraphPad Software Inc., La Jolla, CA, United States) was used for the analysis. Differences between two groups were analyzed by unpaired Student’s t-test, and comparisons between groups were established by one-way analysis of variance (ANOVA) with the least significant difference test. Differences with *p-*values less than 0.05 and 0.01 were considered significant and extremely significant, respectively. * denotes comparisons vs. the control group (*p* < 0.05). # indicates significance among different treatment groups with the model group (*p* < 0.05).

## 3 Results

### 3.1 Prediction of the therapeutic mechanism of POEE that mitigates atherosclerosis

**FIGURE 2:**
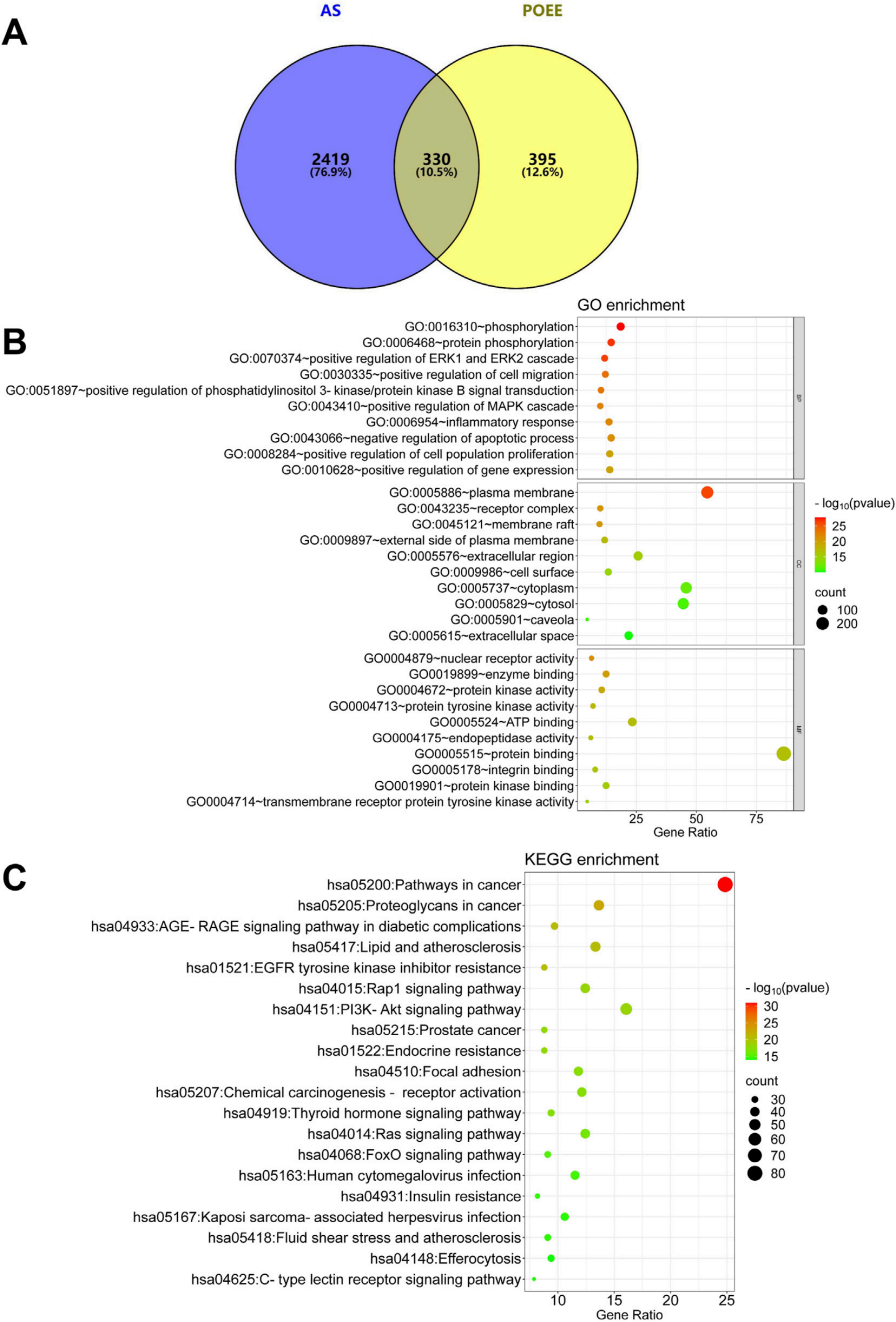
Analysis results of network pharmacology. **(A)** Intersection of CHD targets and compound targets of POEE; **(B)** GO enrichment analysis; **(C)** KEGG pathway enrichment analysis. Bubble size indicates the number of targets within the pathway, and bubble color signifies the *p*-value’s magnitude. The more red the color, the smaller the *p*-value.

First, the contents of flavonoids, alkaloids, and amino acids in POEE were determined to be 6.58, 2.17, and 86.54 mg/g DW, respectively. Subsequently, UPLC-Q-TOF-MS was used to analyze the chemical components of POEE, and Supplementary Figure S1 displays the total ion chromatogram (TIC) of POEE. A total of 822 compounds were identified. Based on the component detection, the TCMSP database, and published research studies, 66 pharmacologically active compounds were selected and categorized into 10 classes: flavonoids (24), terpenoids (16), phenols (6), alkaloids (4), quinones (3), esters (3), steroids (3), lignans (2), organic acids (1), and others (4) (Table 1). A total of 725 targets of POEE’s active components were identified using SwissTarget Prediction and DrugBank (Supplementary Table S1). In addition, 2,749 AS-related targets were obtained from the GeneCards, OMIM, DrugBank, DisGeNET, and GEO databases (Supplementary Table S2). A total of 330 targets were obtained for the treatment of AS after intersection (Figure 2A; Supplementary Table S3). Bioinformatics analysis revealed that the main BPs enriched by POEE intervention include phosphorylation, inflammatory response, and negative regulation of the apoptotic process; the CCs include the plasma membrane, lipid rafts, and cytoplasm; and the MFs include the nuclear receptor, enzyme binding, and protein kinase activity (Figure 2B). POEE is primarily involved in pathways such as lipid metabolism, AS, and the Rap1 and PI3K-Akt signaling pathways during AS development (Figure 2C). The results of the GO and KEGG enrichment analyses are shown in Supplementary Table S4. The lipid pathway is most closely related to AS. Potential targets for improving AS with POEE are marked with five-pointed red stars (Supplementary Figure S2). The key molecules ABCA1, ABCG1, CD36, and MSR1 were selected for subsequent validation.

**TABLE 1:** Active compounds screened from POEE by UPLC-Q-TOF-MS/MS and TCMSP.

No.	Compound	OB	Caco-2	DL	Class
C01	Betaine	24.80	0.36	0.55	Others
C02	Cianidanol	54.82	−0.03	0.24	Flavonoids
C03	(+)-Sparteine	61.89	1.41	0.21	Alkaloids
C04	N-trans-p-coumaroyloctopamine	78.46	0.11	0.23	Phenols
C05	Myricetin*	13.75	−0.15	0.31	Flavonoids
C06	Kaempferol	41.88	0.26	0.24	Flavonoids
C07	Rutin*	16.00	3.20	0.68	Flavonoids
C08	p-Hydroxy-N-(p-hydroxyphenethyl)-cinnamamide	85.63	0.69	0.20	Phenols
C09	Luteolin	36.16	0.19	0.25	Flavonoids
C10	Moupinamide	86.71	0.54	0.26	Phenols
C11	(+)-Syringaresinol*	3.29	0.47	0.72	Lignans
C12	Galangin	45.55	0.54	0.20	Flavonoids
C13	Ginkgolic acid (C15:1)	20.18	1.01	0.31	Phenols
C14	Quercetin	46.43	0.05	0.28	Flavonoids
C15	Morin	46.22	0.00	0.27	Flavonoids
C16	Wedelolactone	49.60	0.32	0.47	Flavonoids
C17	Arctigenin*	8.05	0.67	0.44	Lignans
C18	Kanzonol C*	1.02	0.80	0.45	Flavonoids
C19	Fisetin	52.59	0.20	0.24	Flavonoids
C20	Aloe-emodin	83.37	−0.11	0.24	Quinones
C21	Isorhamnetin	49.60	0.30	0.30	Flavonoids
C22	Diosmetin	31.14	0.46	0.27	Flavonoids
C23	Amentoflavone*	2.95	−0.30	0.64	Flavonoids
C24	Apigenin	23.06	0.43	0.21	Flavonoids
C25	Aurantiamide	45.76	0.12	0.43	Alkaloids
C26	Glabrone	52.51	0.59	0.50	Flavonoids
C27	6-Hydroxyapigenin*	18.97	0.30	0.24	Flavonoids
C28	Rubiadin	25.01	0.48	0.20	Quinones
C29	Glycitein	50.47	0.56	0.23	Flavonoids
C30	Emodin	24.39	0.22	0.23	Quinones
C31	Piperine	42.52	1.12	0.23	Alkaloids
C32	Aurantiamide acetate	58.02	0.32	0.52	Alkaloids
C33	Quillaic acid*	13.06	−0.27	0.72	Terpenoids
C34	Naringenin	59.29	0.28	0.21	Flavonoids
C35	Pseudobaptigenin	70.11	0.57	0.31	Flavonoids
C36	Hinokiflavone*	2.51	−0.07	0.61	Flavonoids
C37	Isoimperatorin	45.46	0.97	0.22	Others
C38	Bilobetin*	7.26	−0.13	0.63	Flavonoids
C39	β-Cryptoxanthin	25.16	1.84	0.57	Terpenoids
C40	Ginkgolic acid (C13:1)*	16.99	1.01	0.23	Phenols
C41	Euscaphic acid*	17.31	−0.21	0.71	Terpenoids
C42	Echinocystic acid	24.43	0.09	0.73	Terpenoids
C43	Methoxyluteolin	26.75	0.37	0.30	Flavonoids
C44	Tectorigenin	28.40	0.52	0.26	Flavonoids
C45	Dehydrotumulosic acid	31.07	0.05	0.81	Terpenoids
C46	18β-Glycyrrhetinic acid	22.05	0.10	0.74	Terpenoids
C47	Poricoic acid A	30.60	−0.13	0.76	Terpenoids
C48	Corosolic acid*	18.55	0.09	0.74	Terpenoids
C49	Linolenic acid ethyl ester	46.10	1.54	0.20	Esters
C50	Pachymic acid	33.62	0.10	0.81	Terpenoids
C51	Kukoamine A*	1.42	−0.20	0.56	Phenols
C52	2-Palmitoylglycerol	26.73	0.39	0.21	Esters
C53	Glyceryl monooleate	34.13	0.23	0.30	Esters
C54	Abietic acid*	16.45	1.13	0.28	Terpenoids
C55	Eicosapentaenoic acid	45.66	1.34	0.21	Organic acids
C56	Glabrolide*	17.46	0.29	0.61	Terpenoids
C57	Oleanolic acid	29.02	0.59	0.76	Terpenoids
C58	Ursolic acid*	16.77	0.67	0.75	Terpenoids
C59	Betulinic acid	55.37	0.73	0.77	Terpenoids
C60	Tigogenin*	13.16	0.83	0.80	Terpenoids
C61	Ergosterol peroxide	44.39	0.86	0.82	Others
C62	Stigmasterol	43.82	1.44	0.75	Steroids
C63	α-Spinasterol	42.97	1.44	0.75	Others
C64	Lupenone*	11.66	1.48	0.78	Terpenoids
C65	Fucosterol	43.78	1.34	0.76	Steroids
C66	Campesterol	37.58	1.32	0.71	Steroids

Note: * represents the components with pharmacological activity reported in the literature.

POEE, *Portulaca oleracea* L. extract; TCMSP, traditional Chinese medicine systems pharmacology database and analysis platform; OB, oral bioavailability; Caco-2, human Caucasian colon adenocarcinoma; DL, drug likeness.

### 3.2 POEE reduces the formation of foam cells caused by ox-LDL

**FIGURE 3:**
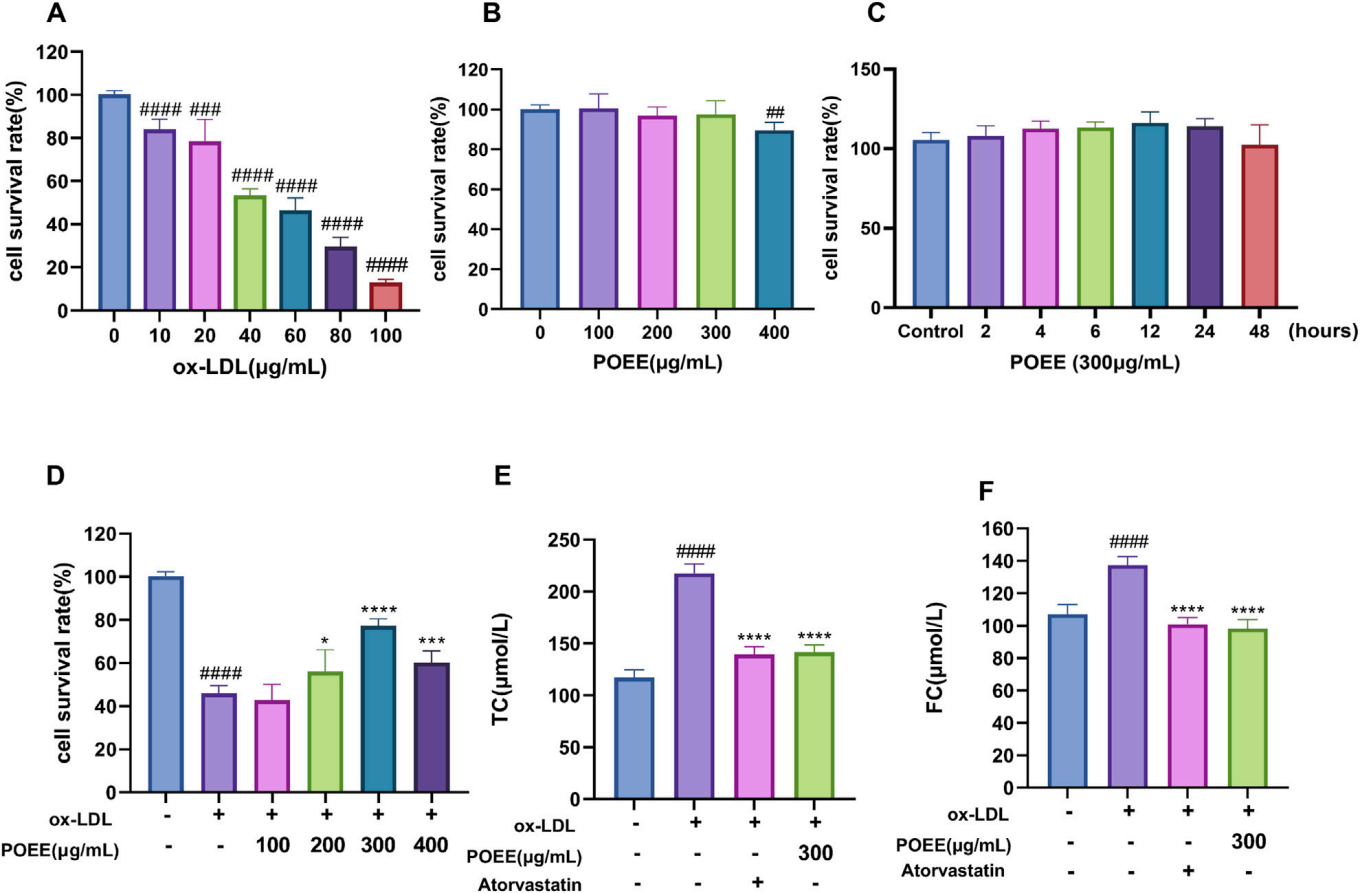
Effect of ox-LDL on foam cell development. **(A)** Proliferation activity of foam cells induced by different ox-LDL treatments. **(B)** Different concentrations of POEE on RAW264.7 cell viability. **(C)** Different treatment times for 0–48 h of POEE (300 μg/mL) on RAW264.7 cell viability. **(D)** Different concentrations of POEE on cell viability induced by ox-LDL (60 μg/mL). **(D)** TC concentrations of foam cells. **(E)** FC concentrations of foam cells. *p < 0.05, ***p < 0.001, ****p < 0.0001 when compared with control, ##p < 0.01, ###p < 0.001, ####p < 0.0001 when compared with model group (n = 6).

We established a macrophage-derived foam cell formation model referring to Zhang C. et al. (2023). The effects of POEE (100, 200, 300, and 400 μg/mL) and ox-LDL (10, 20, 40, 60, 80, and 100 μg/mL) on cell proliferation were confirmed by MTT assay. We noted that POEE at 100–300 μg/mL did not influence cell viability; however, 400 μg/mL POEE inhibited/increased the cell viability (Figure 3A). Different concentrations of ox-LDL 10–100 μg/mL doses reduced the cell activity (Figures 3A, B). The 300 μg/mL POEE treatment did not cause a decrease in the cell activity, and there was no significant difference in the cell activity between the treatment group and the control group at 0–48 h (Figure 3C). Afterward, the influence of POEE on foam cell formation was investigated by administering POEE for 24 h. We observed that POEE (200–400 μg/mL) significantly enhanced the proliferation of ox-LDL (60 μg/mL)-induced injured cells (Figure 3D). In addition, we found that POEE markedly reduced intracellular TC and FC contents, demonstrating an efficacy comparable to that of the positive control drug, atorvastatin (Figures 3D-F). This suggested that POEE effectively prevented the formation of foam cells induced by ox-LDL.

### 3.3 POEE reduced lipid accumulation in foam cells

**FIGURE 4:**
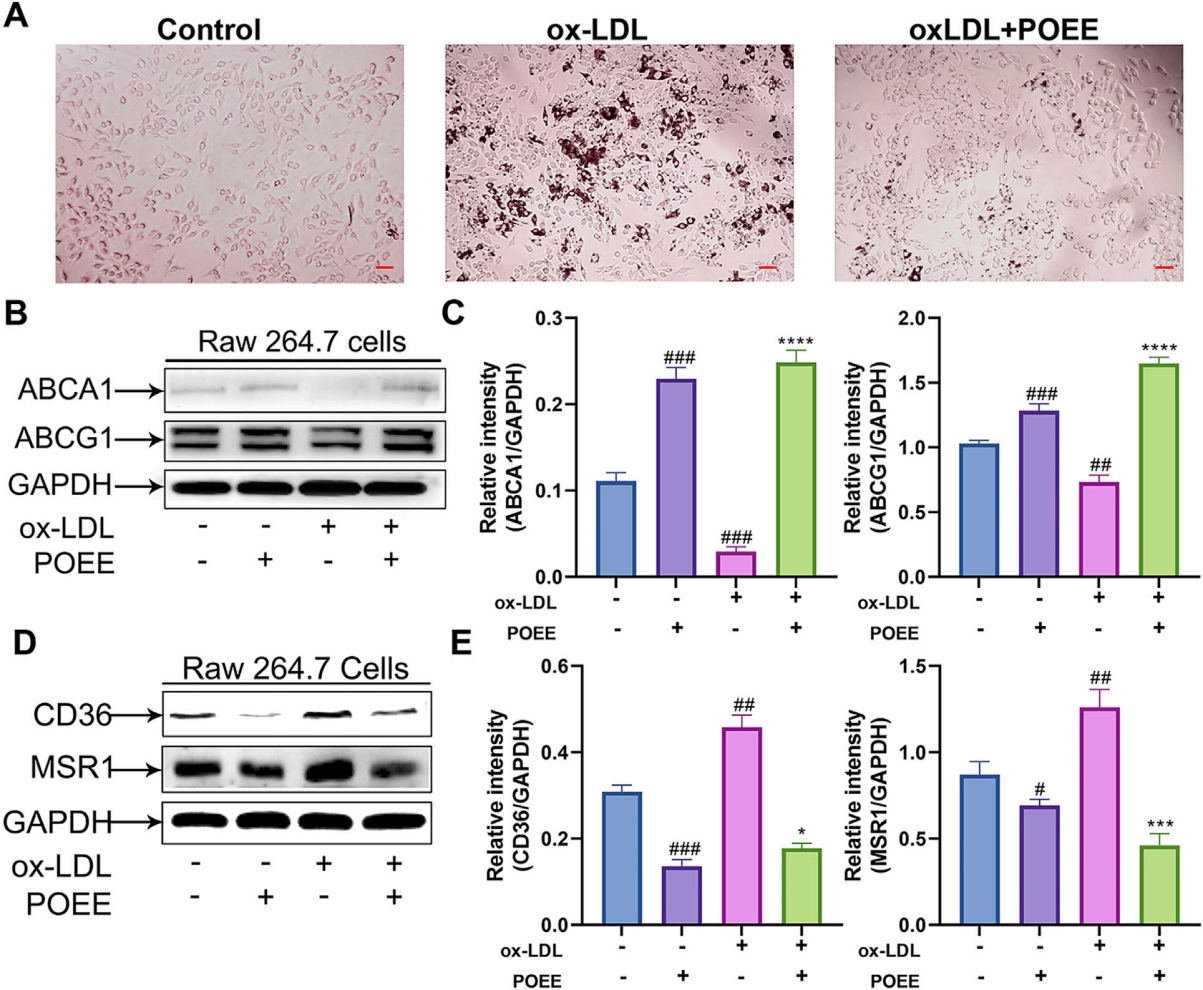
Lipid accumulation in foam cells was alleviated by POEE. **(A)** Foam cells were depicted in images and stained with Oil Red O. (×200 magnification). Scale bar = 20 μm. **(B–E)** Protein expressions of ABCA1, ABCG1, CD36, MSR1, and GAPDH were measured by WB. *p < 0.05, ***p < 0.001, ****p < 0.0001 when compared with control, #p < 0.05, ##p < 0.01, ###p < 0.001 when compared with model group (n = 3).

The ox-LDL induction was performed according to a previous study using ox-LDL. We employed Oil Red O staining to assess lipid accumulation in foam cells. Treatment with POEE markedly reduced lipid deposition induced by ox-LDL in foam cells (Figure 4A). Macrophages clumping together and becoming foam cells is a pathological indicator of AS. Further analysis on the expression of proteins responsible for cholesterol influx (CD36 and MSR1) and efflux (ABCA1 and ABCG1) in macrophage foam cells was performed. Our findings demonstrated that POEE significantly upregulated the expressions of cholesterol efflux transporters, such as ABCA1 and ABCG1, while concurrently downregulating the expressions of CD36 and MSR1, thereby inhibiting cholesterol uptake (Figures 4B–E). The results indicated that POEE boosted cholesterol removal and lowered cholesterol absorption, helping reduce lipid accumulation in the formation of foam cells.

### 3.4 POEE alleviated AS progression in rats

**FIGURE 5:**
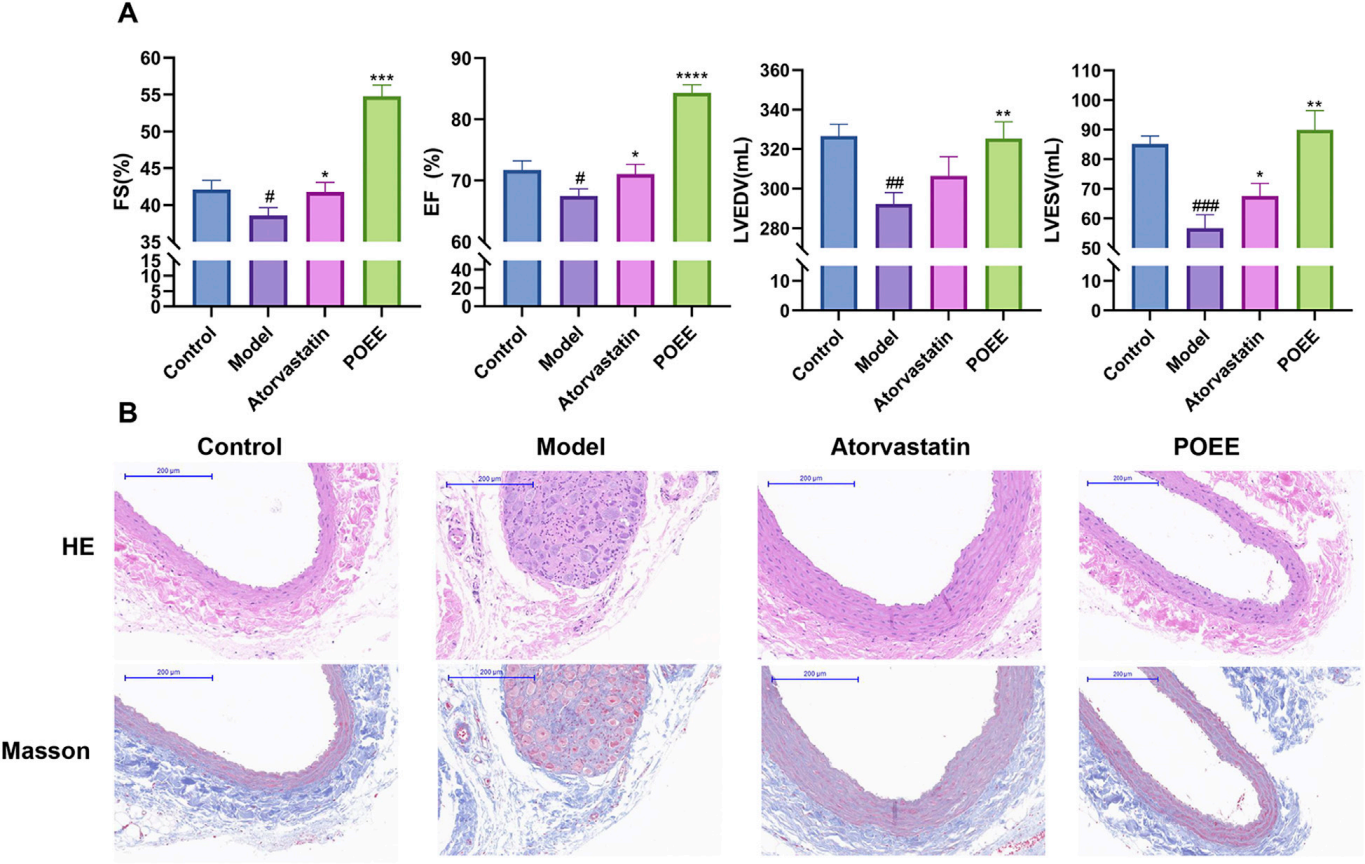
POEE alleviated atherosclerosis progression in S-D rats. **(A)** Cardiac function indexes of rats in each group. **(B)** Images showing HE and Masson staining of the carotid artery. *p < 0.05, **p < 0.01, ***p < 0.001, ****p < 0.0001 when compared with control, #p < 0.05, ##p < 0.01, ###p < 0.001 when compared with model group (n = 3).

We established an animal model of AS using SD rats to confirm the effects of POEE on AS. After 4 weeks on HFD, cardiac ultrasound results show that POEE enhanced the FS, EF, LVESV, and LVEDV in the model group compared to that in the vehicle group; improved the level of cardiac hypertrophy; and delayed the disease process compared to that in the model group (Figure 5A).

**FIGURE 6:**
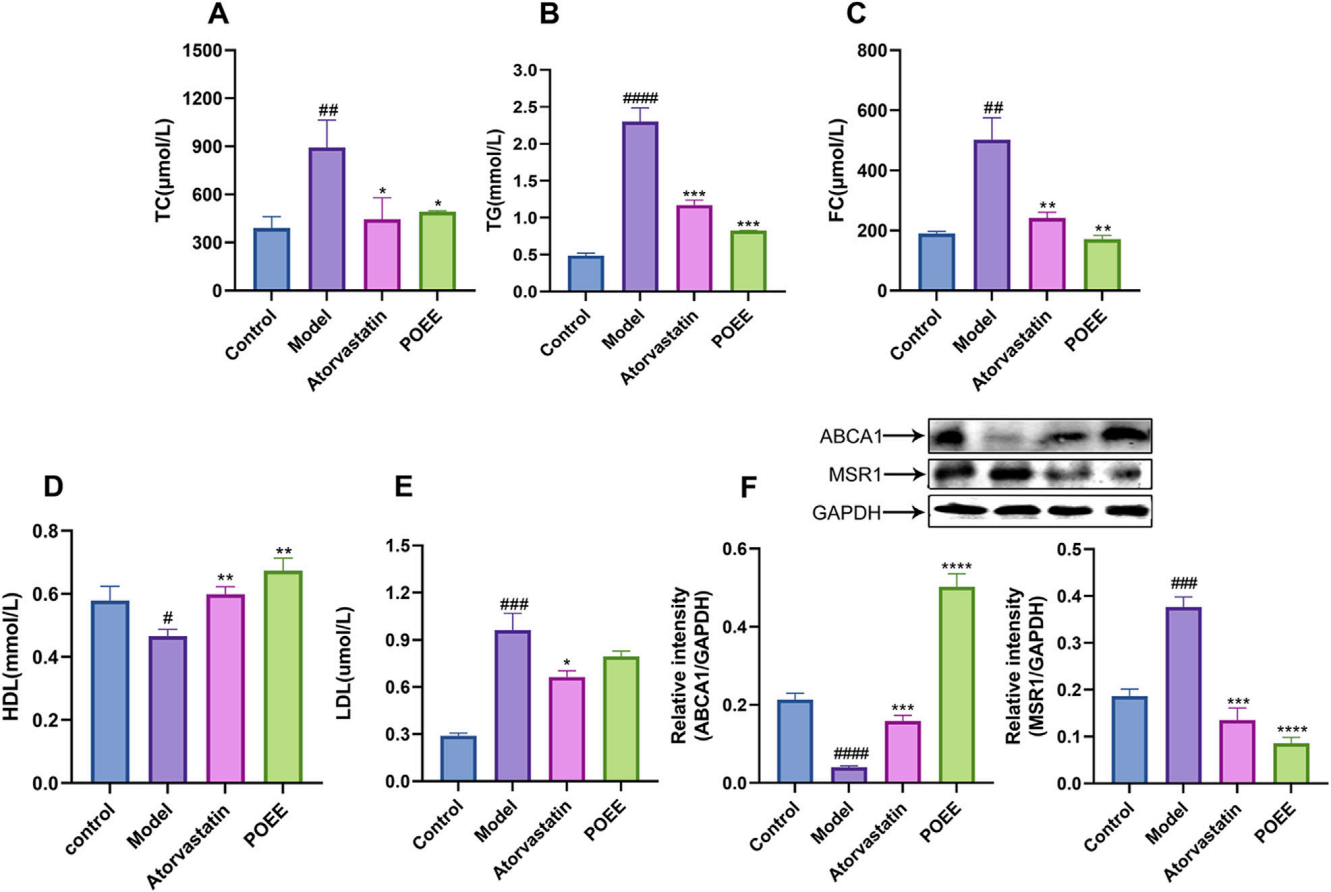
Role of POEE in the progression of atherosclerosis in rats. **(A–E)** Detection of serum levels of TG, TC, FC, HDL, and LDL. **(F)** Measurement of ABCA1 and MSR1 expression. * p < 0.05, ** p < 0.01, *** p < 0.001 when compared with control, # p < 0.05, ## p < 0.01, ### p < 0.001, #### p < 0.0001 when compared with model group (n = 3).

HE and Masson staining were used to observe the structural damage of the carotid artery. Compared to the control group, the model group showed a disordered vascular elastic membrane, thickened carotid artery intima, and cells filled with fat droplets (Figure 5B). In addition, these histological changes reverted after the administration of atorvastatin or POEE. Notably, the effect of POEE was better than that of atorvastatin. Masson’s staining of the carotid artery validated the increase in collagen content *t* in the POEE group (Figure 5B). In addition, POEE significantly increased the serum HDL level and decreased the levels of TG, TC, FC, and LDL in the atherosclerotic rat models (Figures 6A–E). We further investigated the protein expressions of MSR1 and ABCA1 in the carotid artery of rats treated with POEE using WB. The results showed that POEE significantly increased the expression levels of ABCA1 and decreased the expression levels of MSR1 (Figure 6F). Collectively, POEE alleviates AS progression in rats.

## 4 Discussion

This study characterized 882 components in POEE through the LC–MS technology, with the majority of the compounds having been previously reported in the literature (Li et al., 2024). Subsequently, 66 active components were preliminarily screened using TCMSP, mainly including flavonoids, terpenoids, phenols, and alkaloids. In plants, flavonoids provide defense against biotic and abiotic challenges, while in humans, they help avert degenerative diseases when included in the diet (Shen et al., 2022). They possess general characteristics such as antioxidant, antibacterial, and anti-inflammatory activities (Li et al., 2024). According to previous reports, kaempferol, apigenin, luteolin, myricetin, and quercetin are the main flavonoids found in POEE (Zhu et al., 2010). A recent research reported the anti-atherosclerotic effects of the aforementioned five dietary flavonoids (Salvamani et al., 2014). Modern pharmacological studies have shown that kaempferol alleviates palmitic acid-induced lipid storage through AMPK/mTOR-mediated adipophagy (Varshney et al., 2018). Luteolin reduces body fat storage by promoting the central 5-hydroxytryptamine pathway (Lin et al., 2020). *Portulaca oleracea* and its main component, myricetin, alleviate non-alcoholic fatty liver disease by downregulating and inhibiting prostaglandin-endoperoxide synthase 2 (PTGS2) (He et al., 2021). In addition, alkaloids in *P. oleracea* have been proven to have significant antioxidant and antitumor activities (Li et al., 2024). For instance, aurantiamide and aurantiamide acetate have been reported to possess significant antioxidant activity (Chen et al., 2016; Tamokou et al., 2012). Other research studies have shown that oral intake of the terpenoid component *β*-cryptoxanthin can exert anti-obesity effects, which are related to the inhibition of lipid formation in 3T3-L1 cells by RAR activation (Shirakura et al., 2011). Overall, these compounds contribute effectively to the anti-atherosclerotic properties of POEE. In addition, our network pharmacology study showed that anti-atherosclerotic effects of POEE are most closely related to the lipid and atherosclerosis pathway. It has been reported that during the occurrence of AS, cholesterol efflux in macrophages and lipid accumulation play a key role in this process (Wang et al., 2021). Further validation experiments were guided by the predicted molecular mechanisms.

Cholesterol metabolism-related molecules, including MSR1, CD36, ABCA1, and ABCG1, regulate cholesterol metabolism during macrophage transformation into foam cells (Liu et al., 2024). ABCA1 and ABCG1 are located in the plasma membrane of cells and mediate the outflow of intracellular cholesterol to apolipoprotein A-I (apoA-I) and HDL (Ouimet et al., 2019). A decreased cholesterol excretion leads to foam cell formation, which triggers an inflammatory response and formation of atherosclerotic lesions in the arterial wall (Zhang J. et al., 2023; Wu et al., 2024). The other scavenger receptors are macrophages that ingest ox-LDL and CD36; MSR1 is a scavenger of two primary ox-LDL receptors on the cell membrane, devouring ox-LDL and activating the formation of foam cells (Shen et al., 2018). Our laboratory has focused on cholesterol efflux and uptake and found that an upregulation in ABCA1, ABCG1, and CD36 and downregulation in MSR1 expressions accelerate cholesterol efflux and are beneficial. Therefore, effective removal of excess lipids is necessary for AS prevention. This may be a promising strategy for preventing AS.

*In vitro* experiments showed that POEE could regulate the expressions of ABCA1, ABCG1, MSR1, and CD36 in ox-LDL-induced foam cells. The expressions of MSR1 and CD36 were downregulated, therefore inhibiting the positive feedback of ox-LDL uptake. The upregulation of ABCA1 and ABCG1 expressions promoted positive feedback of ox-LDL excretion. The combination of ABCA1 and ABCG1 can reduce free cholesterol in macrophages and cholesterol accumulation. MSR1, CD36, ABCA1, and ABCG1 are considered to be the main therapeutic targets of AS caused by dysfunction of cholesterol metabolism, and these proteins play a key role in the RCT (Chawla et al., 2001). In the experiment of TC and FC index in cells and Oil Red O staining, the intracellular lipid determination results are consistent. On the other hand, in experimental rats, carotid atherosclerotic tissue WB results are consistent with experimental results of *in vitro* cells. This shows that POEE can adjust lipid content in the cell across the plasma membrane.

In animal experiments, according to the results of HE and Masson staining, the alcohol extract of purslane can significantly reduce the lesion area in carotid artery AS. This prevents the rupture of vulnerable plaques, necrotic core, and the formation of secondary thrombosis. In this study, we found that administration of HFD increases the serum levels of TG, TC, FC, and LDL in rats and causes a rapid decline in HDL. POEE helped mitigate the sharp increase in TG, TC, FC, and LDL levels and significantly increased the HDL level after the intervention. These findings are consistent with those of Wang’s study, who was able to change the lipid content in the serum and eggs of laying hens supplemented with a moderate amount of purslane extract (Wang et al., 2020).

Notably, POEE’s cholesterol-lowering efficacy paralleled that of atorvastatin (Figures 3D,E), yet its mechanism diverges fundamentally from that of statins. While statins inhibit hepatic cholesterol synthesis via HMG-CoA reductase (Schonewille et al., 2016), POEE uniquely targets macrophage lipid handling through ABCA1/ABCG1-mediated cholesterol efflux and CD36 downregulation (Figure 4). Histopathological evidence underscores POEE’s therapeutic potential, including reduced intimal thickening and alleviation of carotid artery pathologies (HE and Masson staining, Figure 5B). In contrast, statins primarily reduce plaque volume without directly modulating the collagen content (Makowski and Botnar, 2013).

In addition, these findings demonstrated the effectiveness of POEE in AS and that it might be a promising target for the disease by adjusting the blood lipid levels and reversing cholesterol’s role in AS protection and treatment. There was a significant negative correlation between macrophage cholesterol flow capacity and the strength of an artery’s intima-media thickness and cardiac function. Lipid peroxide and macrophage cholesterol intake outflow ability can lead to the accumulation of cholesterol in the cell and foam cell formation, during the development of AS in streaks of fat and plaque. Increased aortic sclerosis leads to increased cardiac afterload, leading to centripetal remodeling (Djellouli et al., 2018). Ultrasound evaluation of a rat’s heart in the animal experiments showed that FS, EF, LVESV, and LVEDV of rats treated with POEE were significantly increased compared with untreated rats, indicating that POEE delayed cardiac structural changes and deterioration of cardiac function, inhibited ventricular remodeling, and had a significant cardiac protective effect. Studies have shown that the aorta is a tube that carries blood to surrounding organs and acts as a buffer against the heart’s pulsating pressure and outflow of blood (Sanz et al., 2019). Aortic sclerosis has a low buffering effect on pulsating pressure, thereby resulting in increased left ventricular (LV) afterload and decreased systolic function of the LV. After adjusting for age, blood pressure, and other cardiovascular risk factors, the ventricular systolic index was independently related to left ventricular systolic and diastolic dysfunction. The increases in the aortic stiffness of left ventricular systolic and diastolic dysfunction had a direct impact. Therefore, POEE may reduce the incidence of AS and cardiovascular diseases (Munguia-Realpozo et al., 2024).

This study has several limitations. First, the assessment of POEE’s biosafety was validated only in the RAW264.7 macrophages. Although a 4-week *in vivo* toxicology study showed no significant body weight reduction or mortality in rats at the 1.0 g/kg/day dose, systemic toxicity should be further assessed. Second, regarding POEE’s regulatory network in lipid metabolism, other modulation mechanisms, including LXR-mediated cholesterol efflux, SREBP-controlled lipid synthesis, and PPAR-regulated metabolic clearance pathways, should also be investigated in future studies.

## 5 Conclusion

In this study, key active elements of POEE were discovered and examined, with network pharmacology predicting the possible mechanisms through which POEE mitigates AS. In this study, we demonstrated that POEE could improve the lipid profiles of foam cells and HFD-fed rats, while attenuating the risk of AS. Furthermore, our findings indicated that the improvement in overall RCT by POEE in living organisms could be a mechanism behind its anti-atherosclerotic effects. *In vitro*, POEE upregulated the expressions of ABAC1 and ABCG1 and inhibited those of CD36 and MSR1 to alleviate lipid accumulation. Furthermore, POEE reduced TG, TC, FC, and LDL levels, whereas it elevated HDL levels in rat serum. In addition, POEE increased the expression of ABCA1 and reduced that of MSR1 in the carotid arteries and remarkably alleviated carotid artery pathologies. The study suggests approaches for identifying useful medications or supplements in the treatment of hyperlipidemia or AS.

## Data Availability

The original contributions presented in the study are included in the article/Supplementary Material, further inquiries can be directed to the corresponding authors.

## Glossary

AS: atherosclerosis
BP: biological process
CC: cellular component
CD36: cluster of differentiation 36
DMSO: dimethyl sulfoxide
ESI: electrospray ionization
EF2: ventricular ejection fraction
FS: fractional shortening
FC: free cholesterol
GO: Gene Ontology
HFD: high-fat diet
H&E: hematoxylin and eosin
HDL: high-density lipoprotein
KEGG: Kyoto Encyclopedia of Genes and Genomes
LVESV: left ventricular end-systolic volume
LVEDV: left ventricular end-diastolic volume
MSR1: macrophage scavenger receptor 1
MDA: malondialdehyde
MF: molecular function
MTT: 3-[4,5-dimethylthiazol-2-yl]-2,5 diphenyl tetrazolium bromide
ox-LDL: oxidized low-density lipoprotein
PTGS2: prostaglandin-endoperoxide synthase
RCT: reverse cholesterol transport
S-D: Sprague–Dawley
SDS-PAGE: sodium dodecyl sulfate-polyacrylamide gel electrophoresis
TG: triglyceride
TIC: total ion chromatogram
TCMSP: Traditional Chinese Medicine Systems Pharmacology Database and Analysis Platform
UPLC-MS/MS: ultra-performance liquid chromatography-quadrupole/time-of-flight mass spectrometry
WB: Western blot
LDL: low-density lipoprotein
