# Supplementary material for: Ethanol extract of Portulaca oleracea L. mitigates atherosclerosis through modulation of cholesterol efflux and uptake pathways
Source: Front Pharmacol. 2025 Mar 19;16:1550812. doi: 10.3389/fphar.2025.1550812 (PMC11961987; doi:10.3389/fphar.2025.1550812)
Supplement: Supplementary file 5 [file DataSheet1.docx]

Supplementary_Material

Figures


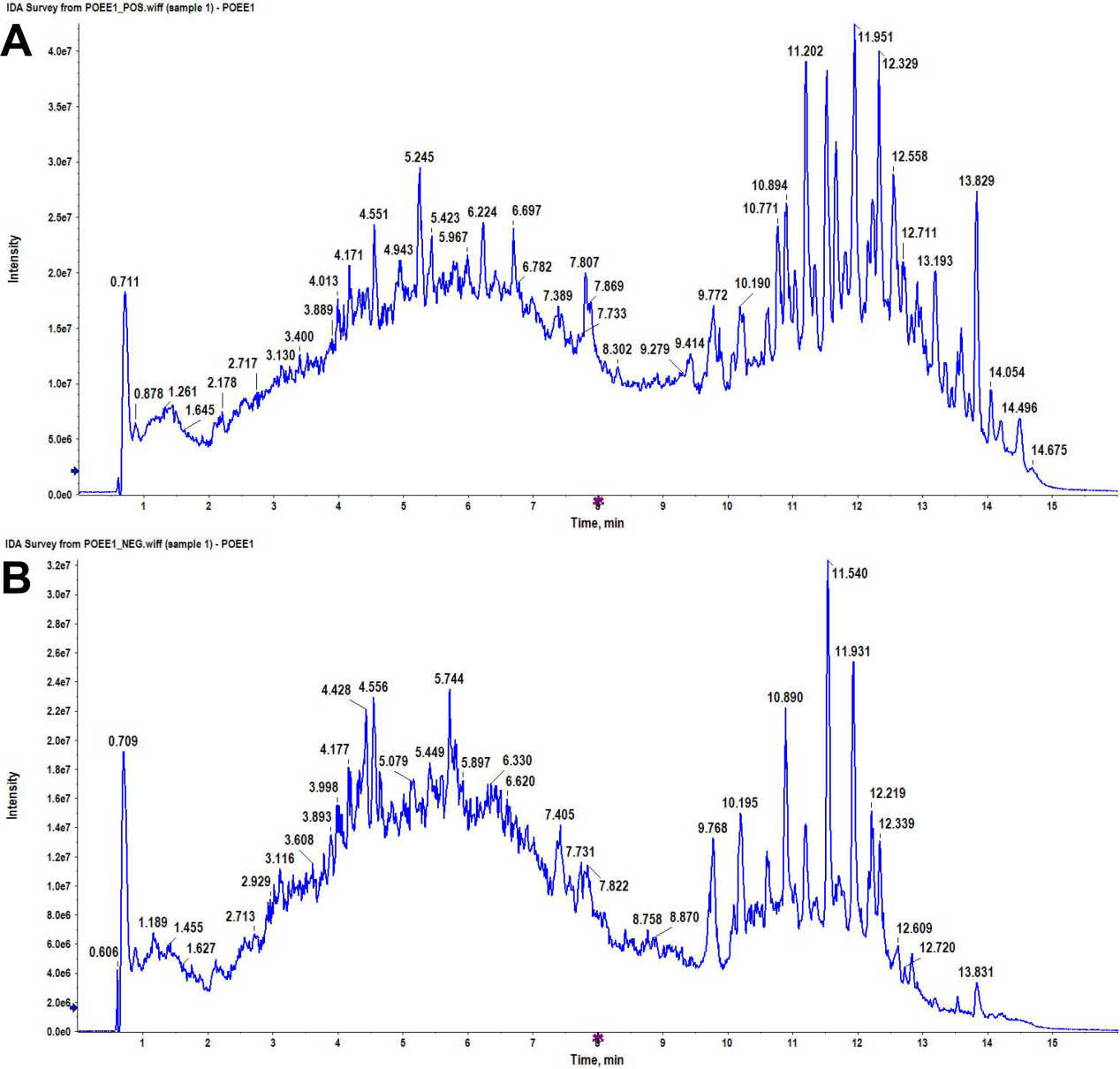


Supplementary Figure 1 The total lon chromatogram of POEE

(A) Positive mode; (B) Negative mode. POEE, *Portulaca oleracea* L. extract.


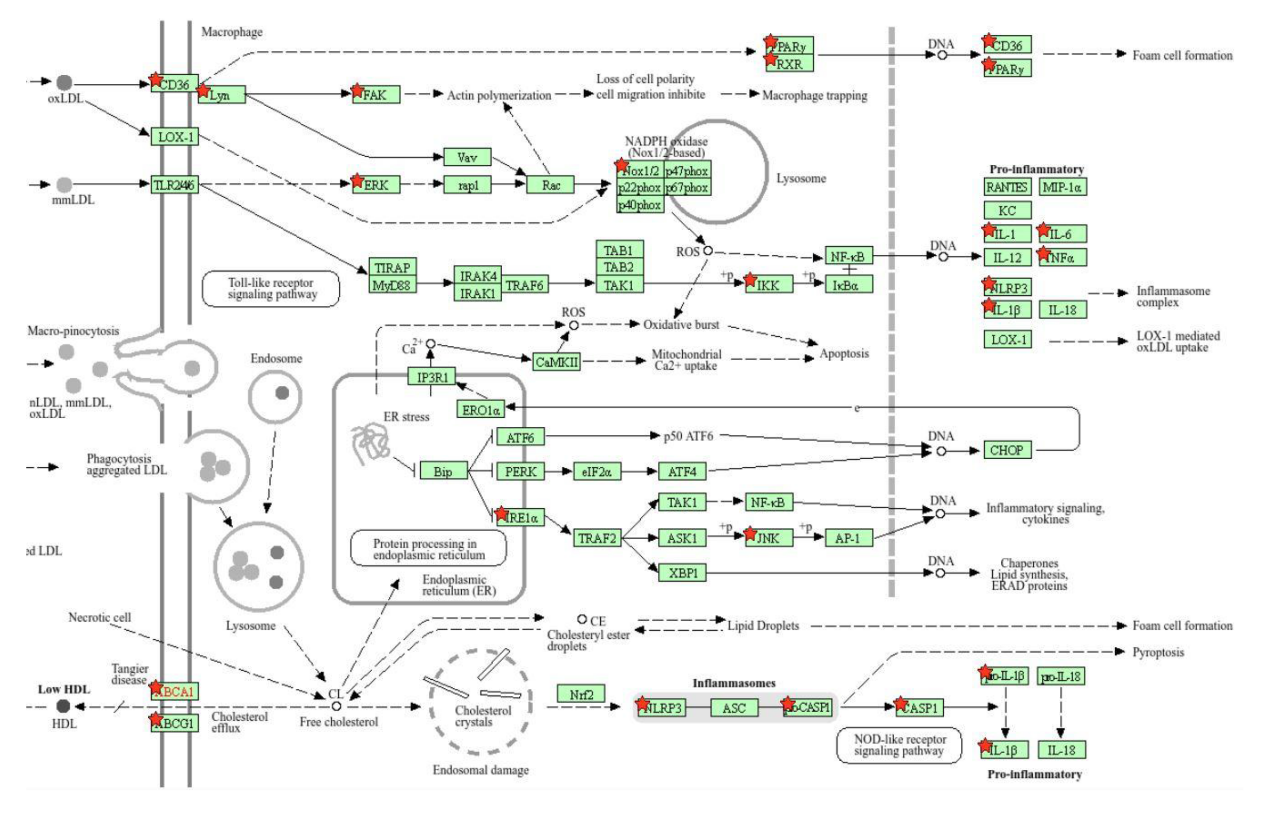


Supplementary Figure 2 Potential targets for improving AS with POEE in pathway lipid and atherosclerosis

AS, atherosclerosis; POEE, Portulaca oleracea L. extract.
